# Diversity and Mechanisms of Action of Plant, Animal, and Human Antimicrobial Peptides

**DOI:** 10.3390/antibiotics13030202

**Published:** 2024-02-21

**Authors:** Galina Satchanska, Slavena Davidova, Alexandra Gergova

**Affiliations:** BioLaboratory-MF-NBU, Department of Natural Sciences, New Bulgarian University, 1618 Sofia, Bulgaria; stdavidova@nbu.bg (S.D.); f104819@students.nbu.bg (A.G.)

**Keywords:** antimicrobial peptides, plant AMPs, animal AMPs, human AMPs, modes of action, benefits and limitations

## Abstract

Antimicrobial peptides (AMPs) are usually made up of fewer than 100 amino acid residues. They are found in many living organisms and are an important factor in those organisms’ innate immune systems. AMPs can be extracted from various living sources, including bacteria, plants, animals, and even humans. They are usually cationic peptides with an amphiphilic structure, which allows them to easily bind and interact with the cellular membranes of viruses, bacteria, fungi, and other pathogens. They can act against both Gram-negative and Gram-positive pathogens and have various modes of action against them. Some attack the pathogens’ membranes, while others target their intracellular organelles, as well as their nucleic acids, proteins, and metabolic pathways. A crucial area of AMP use is related to their ability to help with emerging antibiotic resistance: some AMPs are active against resistant strains and are susceptible to peptide engineering. This review considers AMPs from three key sources—plants, animals, and humans—as well as their modes of action and some AMP sequences.

## 1. Introduction

Antimicrobial peptides, although varying in size, are not usually longer than 100 amino acid residues [1]. They are an important part of the defense and the innate immune systems of many living organisms, such as bacteria, fungi, plants, fish, invertebrates, amphibians, crustaceans, insects, reptiles, mammals, and humans [1]. This review will focus on three in particular—plants, animals, and humans—and their respective modes of action.

Most eukaryotic AMPs are cationic, which accounts for their membrane properties. They have both hydrophilic and lipophilic properties, characterizing them as amphiphilic [2]. Their amphiphilic structure and cationic charge allow them to bind to a negatively charged pathogen cell surface and insert themselves into the membrane, forming pores and channels, which eventually leads to cell death [2]. AMPs can be, according to their biosynthetic origin, ribosomal or enzymatic (NRPS pathway). Ribosomally synthesized AMPs are divided into unmodified ribosomally synthesized peptides and ribosomally synthesized and post-translationally modified peptides (RiPPs). RiPPs are being considered as potential alternatives to conventionally used antibiotics. Among these RiPPs are sactipeptides, which are a small subfamily of peptides. Sactipeptides (sulfur-to-alpha carbon thioether cross-linked peptides) show various biological activities, such as antibacterial and hemolytic properties [3]. Nonribosomal peptide synthetases (NRPSs) are large multimodular enzymes that synthesize a diverse variety of peptides. Many of these are currently used as pharmaceuticals thanks to their antimicrobial activities (penicillin, vancomycin, daptomycin, and echinocandin) and immunosuppressive (cyclosporin) and anticancer compounds (bleomycin) [4].

AMPs use several modes of action when acting on pathogens, some of which target the membrane and some of which focus on intracellular targets, such as nucleic acids, protein synthesis, and repair pathways [5]. Antimicrobial peptides have a broad spectrum of action and are active against both Gram-positive and Gram-negative bacteria, viruses, fungi, and other pathogens [6]. They have been shown to be an effective substitute for antibiotics, as they can act against antibiotic-resistant strains and are naturally found in many living organisms, thus making them easy to access. In Kumar et al.’s review, the authors discussed how antimicrobial peptides can be chemically modified in order to improve their stability against proteolytic digestion while also retaining their strong antibacterial activity [7].

**Figure 1:**
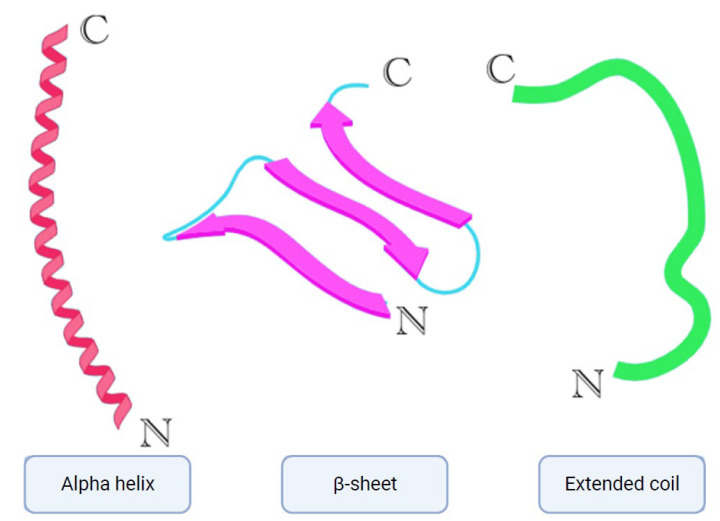
Simplified models of alpha helix, β-sheet, and extended coil structures. Created with biorender.com (accessed on 27 October 2023).

To date, multiple antimicrobial peptides have been isolated from nature, and, because of their diversity, they can also be classified into multiple categories based on their size, structure, modes of action, etc. [7]. AMPs also differ in their activities; some can be active against bacteria—Gram-negative, Gram-positive, or both—while others can be active against fungi or viruses. That said, AMPs can also possess some combination of antibacterial, antifungal, and antiviral activities, or all of these activities at once [7]. Based on their structure, AMPs can be divided into three subclasses [7,8] (Figure 1). The first is AMPs with an alpha helical structure; these are mainly found in animals, specifically frogs and insects [7,8]. Some of the members of this subclass are cathelicidins, including LL-37 (also found in humans, magainins, and aurein peptides. The second subclass is β-sheet AMPs, which include some members of the cathelicidin class, such as protegrins, and other classes, such as defensins and tachyplesins [7,8]. The third subclass of AMPs is known for having an extended coil structure. The members of this subclass consist of mostly cathelicidins (indolicidin) and histatins [7,8]. Notably, cathelicidins can be found across all three of these subclasses.

Cryptic peptides (cryptides) are small bioactive molecules obtained via the degradation of functionally active proteins [9,10,11]. Fesenko et al. found that the winter moss *Phycomitrella patens* produces over 4000 intracellular proteins, and about 500 of them are secreted [10]. The moss was grown under stressful conditions derived from adding the stress hormone methyl jasmonate. As a result, specific proteolysis was induced, leading to the release of peptides with antimicrobial activities. In particular, enzymes are considered to be a reservoir of such cryptic peptides [12]. In their review, the authors described host defense peptides (HDPs) in all canonical classes of enzymes and corresponding hydrolases. Promising anti-biofilm and immunomodulatory activities of human HDPGVF27 on two clinically relevant strains, *Burkholderia multivorans* and *Burkholderia cenocepacia*, were reported by Bosso et al. [13]. *Burkholderia cenocepacia* is an opportunistic pathogen that commonly infects immunocompromised patients. Another study by Ciociola et al. [14] discussed the in vitro and in vivo antifungal activity of a peptide derived from the C-terminus of albumin. This 13-residue peptide can penetrate and accumulate in *Candida albicans* cells, causing gross morphological alterations in the fungus cellular structure. Novel cryptic peptides from PD-L1/2, a type 1 ribosome-inactivating protein (RIP), were reported in a study by Pizzo et al. [15]. The bioactive peptides were found in *Phytolacca dioica* L. The authors described reductions in the biofilm biomass, thickness, and structural components of Gram-negative bacteria.

In this review, we describe three key sources of antimicrobial peptides in detail. We discuss the sources of AMPs in plants and their structures and activities, as they are a crucial source of defense in plants against various pathogens, including viruses, bacteria, and fungi [7,8]. Antimicrobial peptides are of particular importance in plants, as mentioned in Kumar et al.’s review, one of the reasons being their lack of adaptive immunity, meaning B-cell- and T-cell-mediated immunity [7].

The animal kingdom is also an important source of AMPs, and, over the course of this review, we cover the main classes of AMPs found in different groups of animals. Vertebrate animals, contrary to plants, have both innate and adaptive immune systems at their disposal [7]. Nevertheless, some AMPs have been shown to play a critical role in their immune modulation and in reducing inflammation [7,16].

Although humans are mammals, the human body does not produce the same amount of AMPs as other animals. This is discussed in a review by Li et al. [1], where it is mentioned that the only AMP from the cathelicidin family presented in the human body is LL-37, as well as the inability of the human organism to express θ-defensins [1]. In this review, we focus on human antimicrobial peptides, where they are produced, and their activity against pathogens. 

The last section of this review is dedicated to the different modes of action that antimicrobial peptides use to target and eliminate viruses, bacteria, and fungi, with the main modes of action being those that target membranes or focus on intracellular targets.

Table 1 presents the amino acid sequences of some AMPs varying in length and content, most of which were taken from The National Center for Biotechnology’s (NCBI) database [17]. 

## 2. AMPs from Plants

Antimicrobial peptides are very common sources of protection and defense in plants against pathogens, and they are seen in all major organs of plants, including in the seeds and flowers [1,26]. They largely contain cysteine and multiple disulfide bridges, making them compact and durable against high temperatures and chemicals [6,26]. Plant AMPs are divided into groups based on their structures, which include thionins, defensins, hevein-like peptides, knottin-type peptides, α-hairpinins, lipid transfer proteins, snakins, and non-cysteine-rich peptides. These groups differ mainly in their cysteine content and disulfide bridge arrangement [6,27]. Plant cysteine-rich peptides (CRPs) are classified into families based on their sequence similarity; cysteine motifs, which determine their distinctive disulfide bond patterns; and tertiary structure fold. The majority of plant AMPs are cysteine-rich, a feature that enables the formation of multiple disulfide bonds (usually two to six), which contribute to their compact structure and resistance to chemical and proteolytic degradation [28].

### 2.1. Groups of Plant AMPs

Thionins are cationic peptides, found also in plants, with a length of 45 to 48 amino acids, and they contain three to four disulfide bridges [29]. Their activities are primarily antibacterial and antifungal, and they have an effect on both Gram-positive and Gram-negative bacteria [6,29]. 

**Figure 2:**
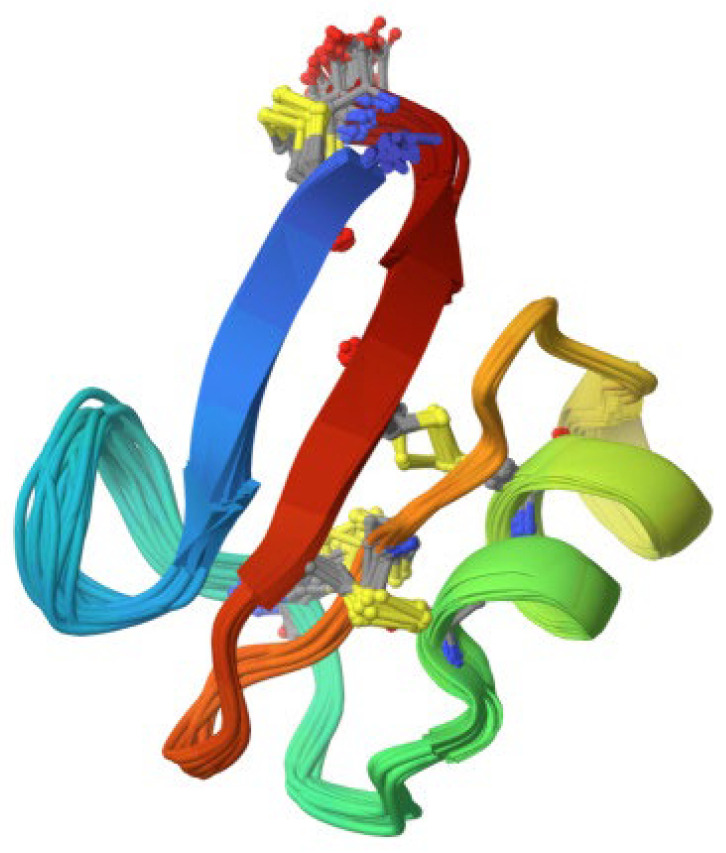
The 3D structure of 4.7 kDa defensin with antifungal activity derived from *Pisum sativum* (source: RCSB PDB/ID 6NOM, Pinheiro-Aguiar et al. [31]).

Plant defensins are also cationic peptides with a similar length of 45 to 54 amino acids, and they contain four to five disulfide bridges [6,30] (Figure 2). They are non-toxic to plant and mammalian cells and, thus, are of interest for their potential use as medicinal agents [30]. Similar to thionins, they have both antibacterial and antifungal properties, and some have even been discovered to have anticancer activities [30]. 

Hevein-like peptides are cationic peptides and can contain from 29 to 45 amino acid residues [6,28]. They contain three to five disulfide bridges and are rich in glycine and aromatic residues [6,28]. Their antifungal activities are due to a chitin-binding domain, which can damage the fungal cell wall; thus, they have a wide range of antifungal activities [27,28]. 

The next class of plant AMPs, knottins, contain 30 residues, including cysteine residues and disulfide bonds [6,28]. Knottins possess a wide range of functions, some of which are hormone-like functions, inhibiting enzyme activity and cytotoxicity [28]. They also have both antiviral and antibacterial activities, but they are toxic to humans due to their unselective contact with cell membranes [6,27,28]. 

α-Hairpinins (stable-like peptides) are rich in lysines and arginines and have a distinctive Cys-motif (XnC1X3C2XnC3X3C4Xn, where X is any amino acid residue, except for cysteine), which forms a helix–loop–helix structure [6,27,32]. They have both antibacterial and antifungal activities, and they bind to DNA and inhibit RNA and protein synthesis by inhibiting trypsin and inactivating ribosome activity [27,32].

Lipid transfer proteins (LTPs) are small, cationic, cysteine-rich proteins made up of around 100 residues [6,27,28]. As their name suggests, they are able to transfer lipids between membranes and thus form pores and cause bacterial and fungal cell death [7]. LTPs are non-specific and can bind to a wide range of lipids, such as phospholipids, fatty acids, and acyl-coenzyme A [28].

Snakins are described as small, cysteine-rich, cationic peptides, similar in structure to thionins and α-hairpinins [28,33]. They have antibacterial (against both Gram-negative and Gram-positive bacteria) and antifungal activities, although their modes of action are not yet fully understood [27,28,33]. 

The last class of AMPs comprises non-cysteine-rich peptides. They have 0 to 1 cysteine residues and are very structurally flexible; their activities are antibacterial (against Gram-negative and -positive bacteria) and antifungal, but they can also exhibit immunostimulatory activities [6,28].

Several AMPs, listed in Table 2, have been expressed in plants with the prospect of clinical and agricultural applications, such as in defense against pathogens and the large-scale and cost-effective production of recombinant AMPs [34,35,36,37,38,39,40].

### 2.2. AMPs Present in Vegetables

In recent years, there have been a lot of experiments proving the existence of AMPs in the vegetables that we consume daily, such as tomatoes, onions, garlic, peppers, and chili peppers, which are covered in the next few paragraphs. Some other examples include LTPs extracted from the leaves of spinach, barley, maize, and sugar beet and from the seeds of cowpea and radish, which have been demonstrated to inhibit phytopathogens, such as *Pseudomonas solanacearum*, *Clavibacter michiganensis*, *Fusarium solani*, *Rhizoctonia solani*, *Trichoderma viride*, and *Cercospora beticola* [41]. 

#### 2.2.1. AMPs in Tomato

In Herbel et al.’s study, the authors mentioned that tomato plants express two members of the snakin peptide family, named snakin-1, SN1 and snakin-2, SN2 [42]. These peptides have been described as being small in molecular weight and having a cationic net charge and six disulfide bonds [42]. Snakins are thought to act through pore formation in the biomembrane of pathogenic cells, but their exact mode of action is not yet fully understood. There is evidence suggesting that SN2 can target the phospholipid membrane of both bacteria and fungi non-specifically and cause the agglomeration of cells. In Herbel et al.’s study, an untreated adult fruiting tomato plant was used to determine SN2 expression levels in a healthy plant. The results of their experiment show that the highest expression of SN2 is found in the leaves and flowers of the tomato plant [42]. They also tested ripe tomato fruits and divided them into skin, pulp, and seeds. The pulp and skin showed moderate expression, whereas, in the seeds, the gene expression level was not detectably higher than in the stem. These results show that every plant organ expresses a distinct amount of SN2 [42]. The authors found a higher quantity of SN2 in the leaves and flowers of the tomato plant than in the shoots, seeds, and roots. Importantly, the study also found that SN2 is expressed constitutively in tomato plants, even if they do not undergo a defense response [42]. Slezina et al.’s study demonstrated that cysteine-rich peptides (CRPs) are also present in tomatoes [43]. The authors described them to be active against the plant pathogen *Clavibacter michiganensis*—Gram-positive bacteria—as well as the fungus *Cryptococcus neoformans*, responsible for fungal meningitis and encephalitis in humans [43].

#### 2.2.2. AMPs in Onion

In Taggar et al.’s study, an AMP, named peptide-Ba49, was isolated from a *Bacillus subtilis* subsp. *spizizenii* strain cultivated from *Allium cepa*, also known as the common onion, which exhibited strong antibacterial activity against *S. aureus* ATCC 25923 [44,45]. The mode of action of this peptide on *S. aureus* was elucidated by the authors to occur through changes in the membrane potential and by triggering the production of reactive oxygen species (ROS) [44,45]. Furthermore, peptide-Ba49 prevented the formation of *S. aureus* biofilms at low concentrations and showed its potential to degrade the mature biofilms of *S. aureus*. Additionally, peptide-Ba49 exhibited intracellular killing potential against *S. aureus* in macrophage cells, and it was found to bolster fibroblast cell migration in a scratch assay at low concentrations, which exhibited the wound healing efficacy of this peptide [44,45].

#### 2.2.3. AMPs in Garlic

In Ezeorba et al.’s review, three peptides were isolated from Laba garlic, namely, F3-3-a, F3-3-b, and F3-3-c peptides, with molecular weights of 693.72 Da, 737.80 Da, and 629.79 Da, respectively [46,47]. F3-3-a was identified as a pentapeptide Tyr-Asn-His-Asn-Phe (YNHNF), F3-3- b was identified as a hexapeptide Trp-Pro-Thr-Ser-Phe-Thr (WPTSFT), and F3-3-c was identified as a hexapeptide Ala-Val-Asp-Arg-Ala-Val (AVDRAV) [46,47]. When examining the antimicrobial activity of the three peptides against *Escherichia coli*, *Staphylococcus aureus*, *Salmonella enteritidis*, and *Bacillus subtilis*, it was found that F3-3-b and F3-3-c had a significant inhibitory activity on the growth of the four bacteria, especially the latter peptide [46,47]. According to the authors, the difference in the amino acid composition and conformation may account for the observed difference in the antimicrobial activity of the peptides [46]. The composition of the hydrophobic amino acids in the peptides was 20%, 50%, and 67% for the F3-3-a, F3-3-b, and F3-3-c peptides, respectively [46]. The presence of the hydrophobic amino acids Val and Ala and the basic amino acid Arg in F3-3-c, which had the highest antimicrobial activity, corroborates previous findings suggesting that Val/Arg residues enhance the antibacterial activity of peptides. The bactericidal action of F3-3-c was demonstrated to cause physical damage to the bacteria cell membrane, thereby initiating the leakage of cellular contents [46].

The same review mentions a new AMP isolated from garlic, AsR416, with a molecular weight of 3799.52 Da, which was found to contain cysteine disulfide bonds, α-helix and β-sheet structures, 1-aspartine, L-histidine, *N*-acetyl-D-glucosamine-6-phosphates, N1-acetyl spermidine, analine, and L-arogenate [47]. AsR416 demonstrated antibacterial activity against Gram-negative bacteria, including the phytopathogens *Agrobacterium tumefaciens*, *Xanthomonas campestris pv. oryzicola*, and *Ralstonia solanacearum*, and Gram-positive bacteria, such as the human pathogens *Bacillus anthrax*, *Bacillus cereus*, and *Bacillus subtilis* and the plant pathogens *Clavibacter fangii* and *Clavibacter michiganensis* [47]. 

Li et al. reported a novel antifungal peptide, named NpRS with nine amino acids (RSLNLLMFR) (Arg, Ser, Leu, Asn, Leu, Leu, Met, Phe, and Arg), obtained from garlic [22]. The peptide was found to significantly inhibit the growth of *Candida albicans.* According to the authors, the mode of action of this peptide is through membrane destruction and interference with ribosome-related pathways and protein synthesis [22]. The resistance gene CDR1 for azole was found to be down-regulated, and drug resistance barely developed in 21 days in a serial passage study. Garlic extracts and garlic oil were also extensively verified to have antifungal activity against *Candida* spp. Garlic oil was constituted by a number of linear sulfur-containing volatile compounds, with diallyl disulfide (DDS) and diallyl trisulfide (DTS) being the most abundant. The authors assessed the antifungal activity of NpRS against *C. albicans* through the minimum inhibition concentration (MIC) and a time-kill kinetics assay [22]. The MIC of NpRS against *C. albicans* was found to be 0.27 mM. The time-kill kinetics assay was performed with fungal strains to determine the mode of action of NpRS on the growth of *C. albicans*. At certain time intervals, aliquots were taken and determined by OD600. According to the results, NpRS exhibited significant fungicide activity (*p* < 0.05) and extended the lag phase of *C. albicans* at the MIC concentration [22]. 

#### 2.2.4. AMPs in Chili Pepper

The *Capsicum* genus is known to produce at least 10 known antimicrobial peptides, including γ-Thionin, found in *C. chinense* [23]. AMPs such as LTPs and defensins were found in the seeds of *C. annuum* [48]. The results indicated that three protein fractions of chili pepper seeds display antifungal activities against different fungi. An inhibitory effect of F1, F2, and F3 fractions on the growth of all fungi tested was observed at concentrations of 70 and 150 µg mL^−1^. A notable inhibitory effect, mainly of the F3 fraction, was also observed on the growth of *S. cerevisiae* yeast at concentrations of 70 and 150 µg mL^−1^, demonstrating 70% and 100% inhibition, respectively [48]. The authors tested the F3 fraction against the yeasts *S. cerevisiae*, *C. guilliermondii*, *C. parapsilosis*, *K. marxiannus*, *P. membranifaciens*, *C. tropicalis*, and *C. albicans*. The IC_50_ value for *C. albicans*, *C. guilliermondii*, *K. marxiannus*, and *P. membranifaciens*, for example, could be observed at a concentration of <16 µg mL^−1^. The growth inhibition of *S. cerevisiae* was observed at a concentration of <32 µg mL^−1^ and for the yeasts *C. parapsilosis* and *C. tropicalis* at <64 µg mL^−1^ [48]. 

In Santos et al.’s study, after protein extraction from the fruit of *Capsicum chinense*, different fractions were obtained, named F1 to F10 [49]. Peptides in the F4 and F5 fractions were sequenced, revealing similarity with plant antimicrobial peptides such as non-specific lipid transfer proteins and defensin-like peptides. The F4 and F5 fractions presented strong antimycotic activity against the phytopathogenic fungi *Fusarium solani* and *Fusarium oxysporum*; they had toxic effects on them, leading to membrane permeabilization, an increase in endogenous reactive oxygen species, the activation of metacaspase, and the loss of mitochondrial function [49].

Table 3 contains different types of plant AMPs and their modes of action against human- and food-borne pathogens.

As shown in Table 3, plant peptides are active against pronounced pathogens such as *E. coli*, *S. aureus*, *S. enteridis*, *C. albicans*, *C. troppicals*, and *C. parapsilosis*. Besides the cell membrane disruption, membrane pore formation, agglomeration, and ROS production of pathogens, it is also worth highlighting the blocking of their biofilm formation or dysfunction by AMPs.

Apart from plant AMPs, other plant-derived compounds include polyphenolic extracts, which also possess antimicrobial properties such as antibiofilm activity against *C. jejuni*, as reported by Elgamoudi et al. [50]. Polyphenol-rich cranberry and other berry extracts have been found to have a strong antibiofilm effect on dual-species *Streptococcus mutans*–*Candida albicans* biofilms and sole *Streptococcus mutans* biofilms [50]. Polyphenols have a high ability to bind to proline-rich peptides; however, the interaction between these compounds present in plants is still not fully understood [51]. Pharmacokinetics, bioavailability, absorption, and metabolism are thought to be affected [51]. The authors concluded that a synergism between polyphenols and proline-rich peptides (PRP) can be achieved by controlling PRP’s gene expression.

The antibacterial activity of different plants against a Gram-positive *B. subtilis* and Gram-negative *E. coli*, as shown in Table 4, is due to the presence of AMPs or polyphenols in plants demonstrating strong activities against various pathogens. The highest antimicrobial action on *B. subtilis* was demonstrated by orange skin onion (27 mm), red skin onion (25 mm), and cayenne pepper (24 mm), and the highest antimicrobial action on *E. coli* was demonstrated by garlic (30 mm) and cayenne pepper (25 mm). 

## 3. AMPs from Animals

AMPs are widely observed in animals, with the defensin and cathelicidin families being the most common. Cathelicidins are positively charged, amphipathic AMPs, and they vary in size and structure [1,53]. Defensins have been proven to be an important part of the animal defense system [1,54,55]. They are cationic and made up of around 29 to 42 amino acids and three pairs of intramolecular disulfide bonds [27,54,55]. Defensins are divided into three groups based on the position of the disulfide bonds, those being α-defensins, β-defensins, and θ-defensins [1,54,55].

AMPs in invertebrates are extremely important, as these organisms lack an adaptive immune response [6]. Some of the main classes of AMPs found in invertebrates are in insects (defensins and cecropins), molluscs and nematodes (defensins), horseshoe crabs (big defensins), invertebrates (β-defensins), and crustaceans (crustins) [6]. Crustins are cationic cysteine-rich peptides that form a tightly packed structure [27]. They have an N-terminal multidomain, which is rich in glycine, cysteine, and proline, and a C-terminal with four disulfide bridges [27]. According to Matos and Rosa, crustins are divided into four classes: Types I to IV. Type I crustins are mainly active against Gram-positive bacteria, while Type II display a broader antibacterial spectrum against Gram-positive and Gram-negative bacteria. Type III and IV crustins are active against both Gram-negative and Gram-positive bacteria. Besides their direct antibacterial effect, crustins display agglutinating properties and can bind to bacterial lipopolysaccharides (LPSs) and lipoteichoic acid (LTA). Interestingly, Type I crustins accumulate in damaged tissues, probably playing a role in tissue regeneration. Crustins are predominantly expressed in hemocytes and are released into the plasma in response to infection and tissue damage. Recently, Type IIa crustins (crusFpau) isolated from the pink shrimp *Farfantepenaeus paulensis* were reported [56]. Saucedo-Vazquez et al. reported marine arthropods to be a promising source of AMPs [57]. Among the broad AMP diversity, the following are the most important: callinectin (*Callinectes sapidus*), armadillidin (*Armadillidium vulgare*), homarin (*Homarus americanus*), anti-LPS factor (many families in the order Decapoda), scygonadin (*Scylla serrata*), penaeidin (*Penaeus monoceros*), hyastatin and arasin (*Hyas arneus*), stylicin (*Penaeus japonicus*), and scyreprocin (*Scylla paramamosaim*) [57]. There has been a recent discovery of a novel AMP in marine worms inhabiting contrasted habitats. The identified AMPs are alvinellacin (ALV), arenicin (ARE), and polaricin (POL—the novel AMP). All three of the AMPs show bactericidal activity against the bacteria typical of the habitat [58].

Insects have also been described to have a large amount of AMPs, such as cecropins, which, again, have been found to have broad activity against Gram-negative and -positive bacteria and some fungi. Other classes of insect AMPs include defensins, proline-rich peptides, and attacins [1,59].

Vertebrate AMPs largely vary in size (15–200 residues) and are present in fish, amphibians, birds, and mammals [6]. AMPs found in fish include β-defensins, cathelicidins, hepcidins, histone-derived peptides, and piscidins [27]. Cathelicidins are secreted by the secretory granules of immune cells and activate when cleaved [6,53]. After being activated, they permeabilize lipid membranes and can act against Gram-positive and -negative bacteria [6,53,60]. Fish β-defensins, containing six cysteine motifs, have antibacterial activity and act against fish-specific viruses. Fish hepcidins are described as cysteine-rich hormones, similar to human hepcidin, with a hairpin structure linked via four disulfide bonds. They regulate iron homeostasis and demonstrate antibacterial activity against both Gram-negative and -positive bacteria [6]. Hepcidins are grouped into HAMP1 and HAMP2, and they act against fish pathogens and induce the internalization and degradation of ferroportin [27]. Piscidins are described as linear amphipathic AMPs, which have a histidine residue and an α-helix that interact with lipid bilayers. Based on their biological activity, amino acid sequence, and length, they are classified into piscidins 1–7 [27]. 

**Figure 3:**
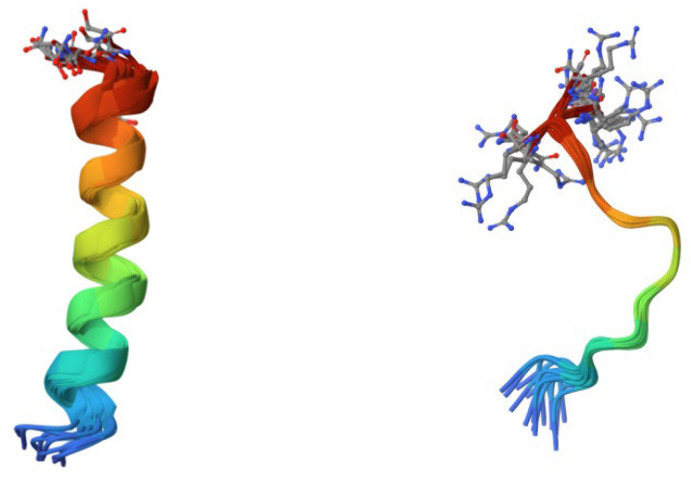
On the left side, the 3D structure of a 23-residue antibiotic magainin isolated from *Xenopus laevis* (source: RCSB PDB/ID 2MAG, Gesell et al., 1997 [62]). On the right side, the 3D structure of an unusually rich in tryptophan bovine antimicrobial peptide indolicidin (source: RCSB PDB/ID 1G89, Rozek et al., 2000 [63]).

The main sources of AMPs in amphibians are frogs and toads [1]. The most common classes that have been observed in them are magainins and cancrins, which have antibacterial activity against both Gram-negative and -positive bacteria and even some fungi [1] (Figure 3). AMPs secreted from frog skin exert potent activity against antibiotic-resistant bacteria, protozoa, yeasts, and fungi by permeating and destroying the plasma membrane and inactivating intracellular targets. Since they do not bind to a specific receptor, those AMPs are less likely to induce resistance mechanisms. Currently, the best known amphibian AMPs are esculentins, brevinins, ranacyclins, ranatuerins, nigrocin-2, magainins, dermaseptins, bombinins, temporins, japonicins-1 and -2, and palustrin-2 [61]. 

Reptile and avian AMPs have been described to be cathelicidins and defensins [6]. A cathelicidin named OH-CATH is a peptide secreted from a king cobra that is active against bacteria such as *Pseudomonas aeruginosa* and *Enterobacter aerogenes*. Avian cathelicidins in chicken are fowlicidines, which are active against both Gram-negative and -positive bacteria [6]. Besides being described in chicken, cathelicidins or cathelicidin-like peptides have also been described in duck, turkey, pheasant, and quail. Reptile β-defensins were first found in the leukocytes of a European pond turtle TBD-1. Crotamine, pelovaterin, and turtle egg-white protein are other AMPs found in reptiles. Avian β-defensins are found not only in chicken-AvBD-1 but also in ostrich-ostricacins and mallard duck-AvBD2 and -AvBD9 [6].

Mammalian-derived AMPs are mostly from the cathelicidin and defensin classes, but others include platelet antimicrobial proteins, hepcidins, and dermcidins [6]. As mentioned, LL-37 from the cathelicidin family is the most studied and understood cathelicidin [6,53]. It is active against various Gram-negative and -positive bacteria and, if applied on wounds, promotes healing [6,53]. Another member of the cathelicidin family present in mammals is cathelicidin 4, or indolicidin, which is a tryptophan and proline-rich peptide, secreted from bovine neutrophils, and it can act against both Gram-negative and -positive bacteria [6,53] (Figure 3). 

Indolicidin forms pores in the cell membrane and inhibits DNA synthesis [6,53]. Protegrins (PGs) are also from the cathelicidin family; they are secreted from porcine white blood cells and act by increasing membrane permeabilization and inhibiting RNA synthesis [6,53,64]. Mammalian α-defensins are secreted by promyelocytes, neutrophil precursor cells, and Paneth cells [6,55]. α-defensins isolated from a guinea pig’s neutrophils can be used against *S. aureus* and *E. coli*, and those isolated from rabbits have broad activity against Gram-negative and -positive bacteria. Mammalian β-defensins were first isolated from bovine mucosal epithelial cells and have been found to be active against Gram-positive and -negative pathogens [6,55]. The structure of Θ-defensins is different to that of α- and β-defensins, and Θ-defensins are active against *B. anthrax*, *S. aureus*, and *C. albicans* [6]. Table 5 presents the AMP classes found in animals. 

## 4. AMPs from Humans

Humans, similarly to animals, can produce AMPs, with LL-37 being the only member of the cathelicidin family that is present in humans [1,53]. LL-37 is produced by epithelial cells and neutrophils and has many activities, such as antibacterial activity, the regulation of inflammation, and the modulation of cell death, and it has further potential in treating drug-resistant bacterial infections. Although LL-37 is primarily found on newborns’ skin, human AMPs can be found in various parts of the body, including the eyes, ears, skin, respiratory tract and lungs, intestines, and urethra [1,53]. Some AMPs are constantly being produced in the body, whereas others are only produced when there is an infection or inflammation present [1]. AMPs in the gastrointestinal tract include human defensins, cathelicidins, regenerating peptides, and metal-sequestering AMPs [65].

Another class of human AMPs comprises defensins. Human α-defensins HNP1-4 (human neutrophil peptides) have been found to be secreted by neutrophils and HD5-6 in the intestinal tract by Paneth cells [6,66]. The most studied and understood defensin is HNP1, which has antibacterial activity against *E. coli*, *S. aureus*, and *S. epidermis* by inhibiting DNA and protein synthesis. HNP1-3 have been found to be abundant in bone marrow, and they can be detected in leukocytes, the spleen, and the thymus [67]. Human β-defensins (HBD1-4) have been found to be expressed in many parts of the body, including the respiratory, gastrointestinal, and urinary tracts; testis; and keratinocytes [6,66]. Human β-defensin 2 (HBD-2) is primarily found in older adults [1,66], but HBD1-3 have antibacterial activity against Gram-negative bacteria, including *P. aeruginosa* and *E. coli*, and yeasts such as *C. albicans*. HBD-3 also expresses antibacterial activities against Gram-positive bacteria, such as *S. pyogenes* and *S. aureus* [6,66]. θ-defensins are not expressed in humans or primates [6].

Histatins 1, 3, and 5 have been isolated from human saliva. All three histatins have the ability to kill the pathogenic yeast *C. albicans*. Other histatins have also been isolated; however, only histatins 1 and 3 have been gene-encoded, since the others are the cleaved products of these two peptides. The histatin genes are located on chromosome 4, band q13, and they are exclusively expressed in human salivary secretions [67].

Dermcidin is an anionic defense peptide found in human sweat. Unlike human defensins and cathelicidins, which are secreted under inflammatory and injured conditions, dermcidin is constitutively expressed in human sweat. Variants of dermcidin, as well as fragments, have also been detected. The level of dermcidin does not vary between healthy people and infected patients. Dermcidin is thought to be related to other human diseases, such as cancer and atherosclerosis [67].

Human liver-expressed antimicrobial peptide-1 (LEAP-1) was discovered in human blood ultrafiltrate and human urine. LEAP-1 is especially rich in cysteine (32%), leading to four disulfide bonds in a 25-residue peptide. This AMP plays an important role in ferrous use, and single-residue mutations in this molecule are associated with severe juvenile hemochromatosis, a genetic disease of severe iron overload. However, unlike LEAP-1, antimicrobial peptide LEAP-2 is not involved in the regulation of iron use [67].

Many AMPs have been found to be synthesized in female and male reproductive systems. They are important for fertility, and, thus, there is a vast diversity of AMPs in the endometrium, vagina, fallopian tubes, and cervix, as reported by Yarbrough et al. [68]. Likewise, in the male reproductive system, some AMPs that have been reported are human cationic antimicrobial peptides hCAP18, defensins, Bin1b, cystatin C, lactoferrin, lipocalin, seminogelin-derived peptides, and lysozyme [69,70].

Table 6 shows select human AMPs with their structures, proposed targets, and modes of action.

## 5. Modes of Action and Mechanisms

### 5.1. Antiviral AMPs

AMPs that are able to target viruses usually have mechanisms that target both RNA and DNA viruses, but, depending on the way that they act, they can be divided into three groups [6,60,64]. The first one consists of AMPs that target the viral envelope. They act by embedding themselves in the envelope of the virus, thus causing instability and disrupting the mode of action of the virus, an example of which is AMP LL-37 [1,6,60,64]. The second mode of action that AMPs can have against viruses is to prevent them from binding to specific receptors on target cells by binding to them themselves [6,60,64]. For example, defensins have the ability to link with herpes simplex virus glycopeptides and thus prevent the virus from binding to the target cell receptors [6]. The third group of antiviral AMPs comprises those that target internal components by either damaging or blocking certain viral proteins, interfering with viral transcription, targeting the viral nucleocapsid, or preventing the virus from leaving the host cell [6,60,64].

### 5.2. Antibacterial AMPs

Antibacterial AMPs have been found to be mostly cationic and amphipathic, and they act by interacting with negatively charged bacterial membranes, causing instability and disruption [6,71]. They act primarily against Gram-negative bacteria, but some AMPs, such as daptomycin, also have a broad spectrum of action against Gram-positive bacteria. Glycopeptides act by binding to cell wall precursors, inhibiting the formation of peptidoglycan [6,71,72]. AMPs have also been suggested as a treatment strategy against *H. pylori* [73]. Antibacterial AMPs have two major modes of action, either bacterial membrane targeting or non-membrane targeting, although AMPs can use both mechanisms [6,71,72]. 

### 5.3. Antifungal AMPs

Fungi, compared to bacteria, have a cellular wall that mostly consists of chitin. Antifungal AMPs have a similar mode of action to antibacterial AMPs: barrel-stave, toroidal pore, and carpet-like models [6,74,75]. These are further described in the following section. Some examples of antifungal AMPs that use these methods of penetrating the fungal cell wall are LL-37 and dermaseptin. Other than these methods, echinocandins, indolicidin, and buforins can inhibit 1,3-β-glucan and chitin biosynthesis and can also act on other intracellular targets, such as nucleic acids and repair mechanisms [6,74,75]. Alamethicin is an antifungal peptaibol. The term is a combination of the words “peptide”, “α-aminoisobutyrate”, and “amino alcohol”. Peptaibols are mainly made by *Trichoderma* fungi. They contain 5–21 amino acids with a high proportion of non-proteinogenic amino acids, acylated N-terminal residues, and amino alcohols linked to the C-terminal [27].

### 5.4. Membrane-Targeting AMPs

**Figure 4:**
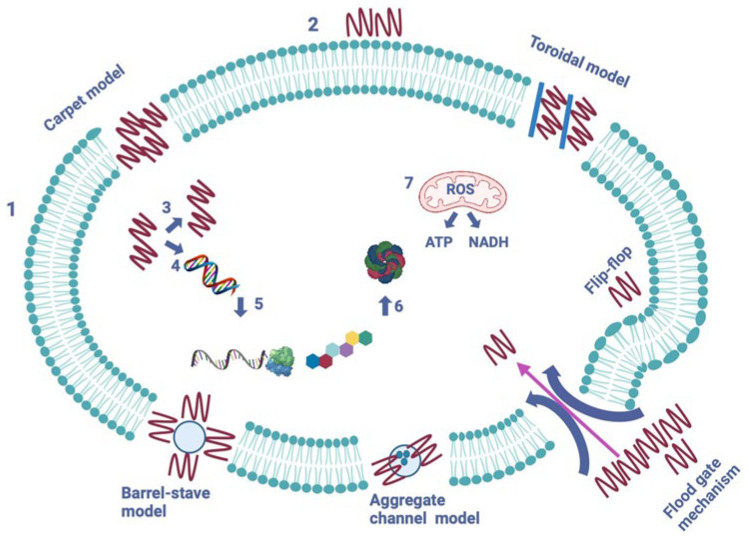
The mechanisms of action of AMPs on a pathogenic cell. Two major types of peptides are pictured—membrane-bound and intracellular active peptides. The outer layer, “1”, is the cytoplasmic membrane of a pathogen cell, and the AMPs are marked as “2”. Membrane-bound peptides are represented by five mechanisms: the barrel-stave, toroidal, carpet, floodgate, and aggregate channel models. The intracellular AMPs are represented by numbers “3” to “7” and show the inhibition of enzymes required for the binding of cell wall structural proteins, DNA and RNA synthesis, ribosomal functions and chaperone protein synthesis, and cellular respiration via ROS (Reactive Oxigen Species) formation, and ATP (Adenosine Triphosphate) and NADH (Nicotinamide Adenine Dinucleotide) Optimized according to Kapil et al., 2020 [72]. Created in biorender.com (accessed on 21 November 2023).

As previously noted, various antibacterial AMPs use a cell-membrane-targeting mechanism, which consists of a positively charged and amphipathic AMP binding to a negatively charged and hydrophobic cell membrane phospholipid, leading to the formation of pores and channels in the cell wall [6,47,60,71]. Thus, to date, five models of cell membrane modes of action have been described, which are the barrel-stave, toroidal-pore, carpet, aggregate channel, and flood gate mechanism models [1,72]. The five models can be seen in Figure 4.

The pores formed in the barrel-stave model lead to cytoplasmic outflow, membrane collapse, permeability, and eventually cell death [6,71] (Figure 4). AMPs that use this mode of action include, but are not limited to, ceratotoxins, protegrins, and alamethicin [1,71]. 

The toroidal-pore model suggests that AMPs can cross the lipid membrane by interacting with the lipid head groups, which causes the lipid bilayer to bend for the peptides to insert themselves into the membrane bilayer and form pores and channels through it. Magainins, protegrins, actinoporins, and melittin are some of the AMPs that use this mode of action [1,6,71].

In the next model, described as the carpet model, AMPs cover the cell membrane’s surface like a carpet and interact with the membrane’s phospholipid head groups [1,6,71]. This eventually leads to high peptide concentrations and the damaging and permeation of the phospholipid bilayer. Some of the AMPs that use this mode of action are cecropins, magainin, and indolicidin [1,6,71].

In the aggregate channel model, AMPs are described to spontaneously form unstructured peptide aggregates, which surround the pathogen’s membrane. This causes the formation of channels and the leakage of cytoplasmic fluid [72]. 

The last model is a recently proposed mechanism, called the floodgate mechanism. During the early stage of the attack, α-helical AMPs form transient toroidal gaps in the pathogen’s cell membrane. It is proposed that AMPs first stress the hydrophobic and electrostatic membrane and that, after the initial attack, they recruit nearby unbound peptides [72]. 

### 5.5. Non-Membrane Targeting

Some AMPs can directly penetrate the cell membrane through endocytosis [1,60]. In this way, they directly act on important bacterial organelles and intracellular proteins, or they can target RNA, DNA, or protein synthesis [1,6,60]. AMPs target the synthesis of nucleic acids and proteins by binding to them and destroying their conformation. Histone-derived AMPs most commonly use this mechanism, and examples include buforin II and indolicidin, the latter of which has activity against both Gram-negative and -positive pathogens. Another way of targeting and inhibiting nucleic acid and protein synthesis is by targeting enzymes and proteins in certain metabolic pathways [1,60]. For example, indolicidin can act on type I DNA topoisomerase and thus inhibit the relaxation of double-stranded DNA. Other AMPs can act on RNA polymerase, DNA gyrase, and other DNA replication-associated proteins [1,6,60]. Other targets of AMPs are the nucleic acid damage repair pathways; they can disrupt damage response pathways and signaling and instead promote cell death and apoptosis. Some peptides can also target ribosomes to inhibit protein synthesis by acting on the translation pathway, usually caused by proline-rich AMPs. PrAMPs can also act on the folding and assembly of proteins by inhibiting the bacterial heat shock protein [1,60].

## 6. Discussion of the Benefits and Limitations of AMPs

Antibiotic-resistant pathogens have become an urgent contemporary problem, as many resistant strains continue to emerge [76]. Antimicrobial peptides can be considered alternative therapeutic agents and may therefore be crucial for the fight against antibiotic resistance [6,77]. As AMPs have many mechanisms of action against pathogens, they have potential benefits not only for clinical practice but also for agricultural biotechnology [26,77]. There is potential with natural AMPs, such as plant-based AMPs, in terms of large-scale production yield, particularly with regard to biotechnological applications of their cultivation [8]. Their clinical efficacy has already been shown [6]. AMPs also have potential as natural food preservatives for food and packaging, as well as a range of health benefits when included in food [29,78]. Interestingly, defatted egg yolk proteins have been found to be a great source of bioactive peptides, which can potentially find application as natural preservatives [79]. 

Along with their advantages, natural AMPs have also been shown to have some limitations, such as poor absorption, poor metabolism, a short half-life, and low permeability, which have all proven to be a challenge in developing drug-alternative AMPs [6,80]. According to multiple articles, some of the main limitations are their poor stability and susceptibly to proteolytic degradation, which can lead to a reduced half-life and limit their possible route of administration when targeting pathogens [27,81]. Additionally, environmental stress can be a contributing factor, as it also leads to the degradation of peptides [82]. While some strategies have been developed to address these challenges, more work still needs to be carried out to ensure the frequent future use of natural AMPs [6,80]. Another disadvantage is that large-scale production and the high cost of production limit their use [27]. Furthermore, as of yet, only a few substances have been approved by regulatory bodies. Those approved include glycopeptides, such as vancomycin and teicoplanin, daptomycin, and polymyxin B, but many more are currently being investigated and tested for their use [6]. To date, many strategies have been developed to increase the stability of AMPs, but these strategies have also been shown to decrease their antimicrobial activity [83]. 

Multiple strategies are currently in development to counter the above AMP limitations, and, so far, various design strategies have been used to improve proteolytic stability, such as sequence modification, cyclization, peptidomimetics, and nanotechnology [6,26,64,79,81]. Many of the plant-based limitations in particular have already been addressed in recent years [26]. Chemical modification has been shown to be the most frequent and easiest way to improve AMP activity and biocompatibility [7,80]. For example, it has been shown that modifying CLEC3A-derived AMPs increases their activity, especially against drug-resistant bacteria [84]. Strategies have been developed to reduce the proteolytic degradation of AMPs, one of which is the development of fully functional mimics of CAMPs (cationic AMPs), which are able to evade proteases [85]. In Cafaro et al.’s study, a peptoid named P13#1 was designed to mimic cathelicidins. The authors reported on the strong biological activities of the peptoid, similar to those of human cathelicidin LL-37, and antimicrobial and anti-inflammatory activities comparable to those of ampicillin and gentamicin, without showing toxicity [85]. A different strategy to overcome the easy degradation of AMPs, implemented by Tortorella et al., was to synthesize the N-glycosylated form of LL-III AMP [82]. The authors concluded that glycosylation did not affect the peptide’s mode of action or biological activity, and it in fact made it more resistant to proteolytic degradation [82]. Another way to tackle the disadvantage of reduced antimicrobial activity when increasing the stability of AMPs was described by He et al. [83]. In their research, they introduced hydrophobic group modifications at the N-terminus of proteolysis-resistant AMP D1 [83]. By end-tagging a Nal, the peptide N1 showed strong antimicrobial activity by damaging bacterial cell membranes and inhibiting the bacterial energy metabolism and retained its stability [83]. Potential approaches to boosting the synthesis of next-generation AMPs to be used as antimicrobial drugs are (i) the modification of the amide bond, (ii) the encapsulation of the peptide in a suitable matrix, (iii) the modification of the amino acid composition, and (iv) the insertion of diastereomers, combined with de novo design strategies [72]. With regard to regulation and approval, for plant-derived AMPs, regulatory bodies have been providing frameworks in recent years, which, for example, has led to the approval of the therapeutic enzyme Elelyso by the FDA [26,86]. Hopefully, in future years, more plant-derived products will be made available for use [26,86].

The overall future use of AMPs is promising [87,88,89]. Recent advancements have brought us closer to their successful implementation [6,86,87]. As studies continue, so too does our ability to increase bioavailability and efficacy, as well as improve the efficiency of production and lower production costs, therefore allowing for greater quantities to be produced [90]. Over the coming decade, we are likely to see further advances in their capabilities, more successful case studies at a trial level, a greater capacity to produce and distribute them, and widespread regulatory approval.

## Figures and Tables

**Table 1:** Amino acid sequences of some AMPs.

AMP	Source	Sequence	Reference
LL-37	Human (*Homo sapiens*)	LLGDFFRKSKEKIGKEFKRIVQRIKDFLRNLVPRTES	[18]
Indolicidin	Cattle (*Bos taurus*)	ILPWKWPWWPWRR-amide	
Crotamine	South American rattlesnake (*Crotalus durissus*)	YKQCHKKGGHCFPKEKICLPPSSDFGKMDCRWRWKCCKKGSG, Cys4-Cys36, Cys11-Cys30, Cys18-Cys37	[18]
Cancrin	Crab-eating frog (*Rana cancrivora*)	GSAQPYKQLHKVVNWDPYG	[19]
Melittin	Honeybee (*Apis mellifera*)	GIGAVLKVLTTGLPALISWIKRKRQQ-NH2	[20]
Buforin II	Asian toad (*Duttaphrynus melanostictus*)	TRSSRAGLQFPVGRVHRLLRK	[21]
HNP-1	Human (*Homo sapiens*)	ACYCRIPACIAGERRYGTCIpYQGRLWAFCC	[18]
HBD-2	Human (*Homo sapiens*)	GIGDPVTCLKSGAICHPVFCPRRYKQIGTCGLPGTKCCKKP	[18]
Protegrin	Pig (*Sus scrofa*)	RGGRLCYCRRRFCVCVGR-amide	[18]
Magainin 2	African clawed frog (*Xenopus laevis*)	GIGKFLHSAKKFGKAFVGEIMNS	[18]
Cecropin A	Cecropia moth (*Hyalophora cecropia*)	KWKLFKKIEKVGQNIRDGIIKAGPAVAVVGQATQIAK-amide	[18]
NpRS	Garlic (*Allium sativum*)	RSLNLLMFR	[22]
SN1	Potato (*Solanum tuberosum*)	MKLFLLTLLLVTLVITPSLIQTTMAGSNFCDSKCKLRCSKAGLADRCLKYCGICCEECKCVPSGTYGNKHECPCYRDKKNSKGKSKCP	[17]
SN2	Tomato (*Solanum lycopersicum*)	MAISKALFASLLLSLLLLEQVQSIQTDQVSSNAISEGADSYKKIDCGGACAARCRLSSRPRLCHRACGTCCARCNCVPPGTSGNTETCPCYASLTTHGNKRKCP	[17]
CC-AMP1	Ghost Pepper (*Capsicum chinense* × *frutescens*)	ZETLDPICMAKCVLKCGKKAWCLTKCIAGCVL	[23]
γ-Purothionin	Wheat (*Triticum turgidum*)	KICRRRSAGF KGPCMSNKNCAQVCQQEGWG GGNCDGPFRRCKCIRQC	[17,24,25]
α-1-Purothionin (precursor)	Bread wheat (*Triticum aestivum*)	MGSKGLKGVMVCLLILGLVL EQVQVEGKSCCRTTLGRNCYNLCRSRGAQK LCSTVCRCKLTSGLSCPKGFPKLALESNSDEPDTIEYCNLGCRSSVCDYMVNAAADDEEM KLYVENCGDACVNFCNGDAGLTSLDA	[17]
Thi2.1	Thale cress (*Arabidopsis thaliana*)	MKGRILILSLLIMSLVMAQVQVEAKICCPSNQARNGYSVCRIRFSKGRCMQVSGCQNSDTCPRGWVNAILENSADATNEHCKLGCETSVCGAMNTLQNSDASEIVNGASEQCAKGCSIFCTKSYVVPPGPPKLL	[17]
Mj-AMP2	Garden four-o’clock (*Mirabilis jalapa*)	MAKVPIAFLKFVIVLILFIAMSGMIEACIGNG GRCNENVGPPYCCSGFCLRQPNQGYGVCRNR	[17]
Lipid transfer protein	Common tobacco (*Nicotiana tabacum*)	MEMVGKIA CFVVLCMVVVAPHAEALSCGQVQSGLAPCLPYLQGRGPLGSCCGGVKGLLGAAKSLSDRKTACTCLKSAANAIKGIDMGKAAGLPGACGVNIPYKISPSTDCSKVQ	[17]
Flower-derived plant defensin 2	*Petunia x hybrida*	MARSICFFAVATLALMLFAAYEAEAATCKAECPTWDGICINKGPCVKCCKAQPEKFTDGHCSKVLRRCLCTKPCATEEATATLANEVKTMAEALVEEDMME	[17]
PmAMP1	Western white pine (*Pinus monticola*)	METKHLAYVMFVLVSLFLAMAQPSQASYFSAWVGPGCNNHNARYNKCGCSNISHNVHGGYEFVYQGQAPTAYNTNNCKGVAQTRFSSNVNQACSNFAWKSVFIQC	[17]
SmAMP2	Chickweed (*Stellaria media*)	MLNMKSFALLMLFATLVGVTIAYDPNGKCGRQYGKCRAGQCCSQYGYCGSGSKYCAHNTPLSEIEPTAAGQCYRGRCSGGLCCSKYGYCGSGPAYCGLGMCQGSCLPDMPNHPAQIQARTEAAQAEAQAEAYNQANEAAQVEAYYQAQTQAQPQVEPAVTKAP	[17]
Crustin	Red swamp crayfish (*Procambarus clarkii*)	MLRVLVLSMLVVAALGHLPRPKPPQPGCNYYCTKPEGPNKGAKYCCGPQFLPLIREEKHNGFCPPPLKDCTRILPPQVCPHDGHCPINQKCCFDTCLDLHTCKPAHFYIN	[17]
Hepicidin HAMP2.3	Gilthead seabream (*Sparus aurata*)	MKTFSVAVAVAIVLTFICLQESSAVSFTEVQELEEPMSNDGPIAAYKEMPEDSWKMGYGSRRWKCRFCCRCCPRMRGCGLCCRF	[17]
Histone-derived, partial	Catla (*Labeo catla*)	MSGRGKTGGKARAKAKTRSSRAGLQFPVGRVHRLLRKGNYAERVGAGAPVYLAAVLEYLTAEILELAGNAARDNKKTRIIP	[17]
Piscidin 2	Schlegel’s black rockfish (*Sebastes schlegelii*)	MRFIMLFLVLSMVVLMAEPGEAFIHHIFGAIKRIFGDKQRDMADQQELDQRAFDRERAFN	[17]
Proline-rich AMP	Green mud crab (*Scylla paramamosain*)	MRLLWLLVALAAVVPAAMPASAGYFPGRPPFPRPFPRPPSRPFPRPPFPGPFPRPYPWR	[17]
Attacin	House fly (*Musca domestica*)	MFTKSIAIIVFLATLAVVNAQFGGSITSNS RGGADVFARLGHQFGDNKRNFGGGVFAAGNTLGGPVTRGAFLSGNADRFGGSLSHSRTDNFGSTFSQKLNANLFQNDKHKLDANAFHSRTNLDNGFKFNTVGGGLDYNHANGHGASVTASRIPQLNMNTVDVTGKANLWK SADRATSLDLTGGVSKNFGG PLDGQTNKHI GVGLSHDF	[17]
Oh-Cath	King cobra (*Ophiophagus hannah*)	MEGFFWKTLLVVGALAIGGTSSLPHKPLTY EEAVDLAVSIYNSKSGEDSLYRLLEAVPPPEWDPLSESNQELNFTIKETVCLVAEERSLEECDFQEDGAI MGCTGYYFFGESPPVLVLTCKPVGEEEEQK QEEGNEEEKEVEKEEKEEDEKDQPRRVKRF KKFFKKLKNSVKKRAKKFFKKPRVIGVSIPF	[17]
TBD-1	European pond turtle (*Emys orbicularis*)	YDLSKNCRLRGGICYIGKCPRRFFRSGSCS RGNVCCLRFG	[17]
Pelovaterin	Chinese soft-shelled turtle (*Pelodiscus sinensis*)	DDTPSSRCGSGGWGPCLPIVDLLCIVHVTV GCSGGFGCCRIG	[17]
avian β-defensin 1	Japanese quail (*Coturnix japonica*)	MKIVYLLFPFILLLAHGAAGSSRDLGKREQ CYRQKGFCAFLKCPSLTIISGKCSRFHVCCKNIWG	[17]
α-defensin 5, Paneth cell-specific	Human (*Homo sapiens*)	MRTIAILAAI LLVALQAQAESLQERADEAT TQKQSGEDNQDLAISFAGNGLSALRTSGSQARATCYCRTG RCATRESLSGVCEISGRLYRLCCR	[17]
θ-defensin-1	Rhesus monkey (*Macaca mulatta*)	RCICTRGFCRCLCRRGVC	[17]

**Table 2:** AMPs expressed in plant hosts and their application and activities against pathogens [26].

AMP	Plant Species	Size in kDa	Application and Activity	Reference
Thi2.1 (thionin)	Tomato (*Lycopersicon esculentum*)	5	Crop protection	[26,34]
Mj-AMP2 (knottin)	Rice (*Oryza sativa*)	80	Resistance to fungal pathogens	[26,35]
Lipid Transfer Proteins (LTPs)	Tobacco (*Nicotina tabacum*)	9	Resistance to pathogens	[26,36]
Petunia floral defensins	Banana (*Musa* spp.)	5	Effective resistance against pathogenic fungal *Fusarium oxysporum*	[26,37]
PmAMP1 (cysteine-rich protein)	Canola (*Brassica napus*)	10.6	Resistance against fungal pathogens (*Leptosphaeria maculans*)	[26,38]
SN-1 (snakin)	Wheat (*Triticum aestivum*)	6.9	Antifungal activity in vitro and enhanced resistance to fungus (*Gaeumannomyces graminis*)	[26,39]
Pro-SmAMP2 (hevein-like peptide)	Potato (*Solanum tuberosum*)	2–6	Crop protection from *Alternaria* sp. and *Fusarium* sp.	[26,40]

**Table 3:** Summary of AMPs expressed in vegetables, as well as their modes of action and activity against bacteria and fungi.

Vegetable AMPs	Mode of Action	Active Against
Tomato (snakin SN2)	Pore formation, agglomeration of cells	*S. cerevisiae*
Onion (Ba-49)	Disruption of the cell membrane, triggering the production of ROS *, preventing the formation of biofilms, and degrading the formation of mature biofilms	*S. aureus*
Garlic (F3-3-a, F3-3-b, F3-3-c)	Disruption of the cell membrane	*E. coli*, *S. aureus*, *Salmonella enteritidis*, *B. subtilis*
Chili pepper (F3 fraction)	Membrane permeabilization, production of ROS	*S. cerevisiae*, *C. guilliermondii*, *C. parapsilosis*, *K. marxiannus*, *P. membranifaciens*, *C. tropicalis*, *C. albicans*

* ROS—Reactive Oxygen Species.

**Table 4:** Antibacterial activity of AMP/polyphenol-containing vegetables (inhibition zone d in mm) against *B. subtilis-* and *E. coli*-type strains [52].

Vegetable/Plant Vegetative Organ	Inhibition Zone d on *B. subtilis* NIBMCC 8752	Inhibition Zone d on *E. coli* NIBMCC 8751
Parsley (leaves)	2	0
Tomato (seeds)	5	0
Cayenne pepper (tissue discs)	24	25
Cayenne pepper (seeds)	7	11
Onion orange skin (mature bulbs)	27	3
Onion red skin (mature bulbs)	25	3
Onion young (fresh bulbs)	0	0
Garlic (mature bulbs)	7	30
Garlic young (fresh bulbs)	2	0

**Table 5:** Different classes of AMPs found in animal hosts. Optimized according to reference [27].

Animals	AMPs	Reference
Mammalians	Cathelicidins Defensins Platelet antimicrobial proteins Dermcidins Hepcidins	[6,27,53]
Reptiles	Defensins Cathelicidins	[6,27]
Fish	β-defensins Cathelicidins Hephecidins (HAMP1 and HAMP2) Histone-derived peptides Piscidins (1–7)	[6,27]
Amphibians	Magainins Cancrins	[1,6,27]
Crustaceans	Crustins	[6,27]

**Table 6:** Select human AMPs, their structures, and proposed targets. Optimized according to reference [67].

AMP	Structure	Size in kDa	Mode of Action/Target
HD-6	β	3–5	Aggregate on bacterial surface
HBD-3	αβ	5.1	Bacterial cell wall (lipid II)
HNP-1	β	3	Bacterial cell wall (lipid II)
LL-37	α	18	Bacterial membranes and/or DNA
Dermcidin	α	9.5	Membrane ion channels
Histatin 5	α	3	Intracellular mitochondria

## Data Availability

The data used during the current study are available from the corresponding author upon request.
